# Gender diversity associated with patterns of brain activation seen in populations that experience childhood stress

**DOI:** 10.3389/fnint.2023.1084748

**Published:** 2023-03-09

**Authors:** Hannah Loso, Bader Chaarani, Sarahjane Locke Dube, Matthew D. Albaugh, Aya Cheaito, Hugh Garavan, Alexandra Potter

**Affiliations:** ^1^Department of Psychological Science, The University of Vermont, Burlington, VT, United States; ^2^Department of Psychiatry, The University of Vermont, Burlington, VT, United States

**Keywords:** fMRI, BOLD (blood oxygenation level dependent) signal, gender diversity, stress, ABCD

## Abstract

**Introduction:**

Stressful childhood experiences are associated with unique brain activity patterns during emotional processing. Specifically, pediatric stress is linked to activation in the insulae, superior temporal and parahippocampal gyri, and the amygdalae, as well as differential activation in the dorsal anterior cingulate cortex when viewing emotional faces. Gender diversity is broadly associated with higher victimization and mental health disparities in children aged 9/10, but whether it is associated with stress-like alterations in brain function (BOLD signal during task-based fMRI) remains unknown. We investigate the functional brain correlates of this relationship to determine if gender-diverse youth show patterns of functional activity during an emotional task consistent with those of other populations that experience heightened stress.

**Methods:**

We used data from the Adolescent Brain Cognitive Development (ABCD)^®^ study. First, we identified a subset of 4,385 participants aged 10/11 years with gender diversity data and quality-controlled fMRI data from the EN-Back (emotional *n*-back) task. The EN-Back is a working memory task that presents emotion faces as well as pictures of places as control stimuli. We regressed BOLD signal associated with emotion faces (faces minus places contrast) on gender diversity. Next, we tested if parental acceptance or youth perceptions of their school environment moderated the relationship between gender diversity and activation in the insulae or fusiform gyrus. Finally, we used structural equation modeling to investigate gender diversity’s association with parental acceptance, perceptions of school environments, internalizing and externalizing problems.

**Results:**

Gender diversity was associated with widespread increases in BOLD signal during the faces condition of the EN-Back task. Youth’s report of parental acceptance and school environment did not moderate the relationship between gender diversity and BOLD signal in the insula or fusiform gyrus. Gender diversity was related to greater parent and school-related stress, which was associated with elevated mental health problems.

**Conclusion:**

Patterns of functional activity were consistent with those reported in prior literature on childhood stress. Gender diversity was associated with increased emotional and behavioral problems, as well as parent and school-related stress. These findings indicate the importance of the home and school environments for supporting the wellbeing of gender diverse youth.

## 1. Introduction

Gender is multifaceted and includes how someone identifies, expresses, and feels about their gender. Gender is not just a categorical identity (e.g., cisgender/transgender or boy/girl/non-binary/agender etc.), but rather a constellation of dimensional constructs (see Table 1 for definitions). Internal dimensions of gender include felt-gender, which describes the extent to which someone feels gender, and contentedness, the degree to which someone is content with their gender. External dimensions of gender include expression, behavior, clothing, or mannerisms aligned (or not) with cultural expectations of femininity or masculinity. All facets of gender are relevant for all people. The degree to which an individual does or doesn’t align with societal expectations based on their sex assigned at birth—gender diversity—can vary independently along each construct. For instance, a person may describe themselves as feeling somewhat like the gender that aligns with their sex assigned at birth (felt gender) while mostly dressing, acting, or choosing activities similar to the gender associated with the opposite sex (gender non-conformity). Gender diverse refers to youth who experience some aspect of gender that does not match society’s stereotypes regarding their sex assigned at birth (American Psychological Association Division 16 and 44, 2015).

**TABLE 1:** Definitions of terms used in this manuscript.

Term	Definition
Gender diverse	Any degree of variation from societally defined expectations or stereotypes regarding male or female gender norms
Gender identity	An individual’s internal definition of their gender
Gender non-conformity	Dressing or acting in a way that does not completely conform to the traditional gender stereotypes that society has ascribed to the individual’s sex assigned at birth
Felt gender	The extent to which one feels like the gender aligned with their sex assigned at birth and like the gender not aligned with their sex assigned at birth
Gender non-contentedness	Not feeling content with the gender aligned with one’s sex assigned at birth
Transgender	An umbrella term that describes someone whose gender identity does not align with their sex assigned at birth
Cisgender	This term describes an individual whose gender identity is wholly aligned with the gender society associates with their sex assigned at birth

Gender minority youth (youth with transgender and/or non-binary identities) face overwhelming rates of discriminatory and harmful legislation, institutional discrimination in schools, the justice system, health systems, and public accommodations (e.g., Greytak et al., 2016; Bortz and Safer, 2018; Kosciw et al., 2020), as well as higher rates of peer and family rejection and victimization (e.g., Landolt et al., 2004; Roberts et al., 2012; Gordon et al., 2018). Subsequently, gender minority youth have elevated mental health problems compared to their peers (e.g., Spivey and Prinstein, 2019; Potter et al., 2021). In fact, over 50% of gender minority youth in a 2021 national survey reported considering suicide (Paley, 2021).

Although recent estimates suggest gender minority youth represent approximately 1.8% of the population (Johns et al., 2019), gender diversity is common. One study found that 27% of adolescents in the California school system reported that their peers would describe their gender expression as non-conforming (Wilson et al., 2017). Potter et al. (2021) found that in the Adolescent Brain Cognitive Development (ABCD) study, 33.2% of youth ages 10–11 (approximately 1/3 of a sample of 4,935 participants across 21 sites in the United States) endorse some gender ratings that are not fully aligned with assigned sex. Further, this dimensional gender diversity in this community sample was associated with increased mental health symptoms. Suggesting that level of gender diversity, regardless of minority identity status, is associated with disproportionate negative health outcomes as early as 10 years of age. Higher levels of distress among gender diverse youth continues through high school. Findings from a study conducted by Lowry et al. (2018), suggest that one dimension of gender diversity, gender non-conformity, was associated with greater feelings of sadness and hopelessness among a group of high school students.

While gender diversity is not the same as a gender minority identity, intersocial stressors from non-conformity with dominant culture can significantly impact health and wellbeing. Indeed, previous literature has shown that youth who do not conform to gender conventions are at higher risk of peer victimization and rejection (e.g., Aspenlieder et al., 2009; Toomey et al., 2012). Utilizing data from the ABCD study, our recent research has found that gender non-conformity was associated with increased family conflict and poorer perceptions of school environment such that greater gender non-conformity was associated with elevated total behavioral and emotional health symptoms, increased family conflict and poorer perceptions of the school environment (Loso et al., 2023). Further, family and school stress significantly mediated the relationship between gender non-conformity and mental health problems. Taken together, emerging literature demonstrates that youth who, even slightly, violate cultural expectations regarding gender, experience more mental health problems, and that positive school and family environments can buffer this relationship.

Heightened distress associated with victimization and discrimination among gender diverse youth can be conceptualized with the minority stress model. The minority stress model was first coined by Ilan Meyer (Meyer, 1995, 2003; Meyer and Dean, 1998) to describe the experiences of individuals in the gay, lesbian and bisexual community and was later adapted for application to transgender and gender non-conforming individuals (Hendricks and Testa, 2012). Meyer defines minority stress as the stress that arises when the experience of an individual in a minority group is in contradiction to the majority culture. Minority stress operates through three major processes to create negative health outcomes. (1) Distal stressors are larger, objective, institutionalized discriminations that do not rely on a person’s perceptions of their oppression, whereas proximal stressors are subjective, individual stressors that are based on how an individual appraises a stressful event. Meyer proposed two distinct forms of proximal stress: (2) expecting to experience victimization or discrimination and (3) internalizing negative societal attitudes related to one’s minority status. Proximal and distal stressors are inextricably linked. For instance, if a gender non-conforming child is bullied at school for the way that they dress and the school does not address the harassment (distal stressors) they may become anxious to go to school due to anticipation (proximal stress) that they will be bullied and victimized by their peers and that school personnel will not protect them. Although the expectation of being bullied is a subjective experience, it is in reaction to a real, external threat and may be an accurate expectation. Although minority stress is defined as stress related to having a minority identity (e.g., transgender, non-binary), a minority stress framework can still inform heightened distress experienced by youth who endorse some level of gender diversity.

Research suggests that minority stress gets “under the skin” and is associated with inflammatory biomarkers and poor physical health outcomes (e.g., McQuillan et al., 2021). A single study comparing clinic-referred transgender adults to a cisgender group found altered amygdala processing that was associated with levels of choline (measured with magnetic resonance spectroscopy; Kiyar et al., 2022). While research on the neurobiology of gender diversity-based minority stress is relatively new, hasn’t yet extended to younger samples, and has only been conducted with individuals with a minority identity (as opposed to some level of gender diversity), there is a deep literature on other forms of childhood stress (e.g., maltreatment, poverty, anxiety disorders). For example, a meta-analysis found that maltreated youth have greater activation in the insulae, superior temporal and parahippocampal gyri when viewing emotional faces compared to non-maltreated peers (Hein and Monk, 2017). Other studies of children who experience stress have shown increased activation in the amygdalae when viewing emotional faces (Etkin and Wager, 2007; Hein and Monk, 2017; Miller et al., 2020). Studies have found a relationship between elevated stress and alterations in the dorsal anterior cingulate cortex (Keding and Herringa, 2016; Weissman et al., 2020), however the direction of these effects are mixed. Taken together, pediatric stress may be detectable in brain regions associated with social perception and cognition (superior temporal gyrus); processing subjective feelings and uncertainty (insula); and processing emotion (amygdalae, OFC).

To our knowledge, previous studies have not examined the relationship between gender diversity, environmental stressors, neurobiology, and mental health in a community sample of younger children. To fill this gap in the literature, we first aimed to investigate if levels of gender diversity among a community sample of early adolescents were associated with patterns of functional brain activation consistent with childhood stress. Based on existing literature and our previous behavioral work, we hypothesized that gender diversity would be associated with greater activation in the insula, superior temporal and parahippocampal gyri, and the amygdalae as well as differential activation in the dorsal anterior cingulate cortex when viewing emotional faces compared to when viewing places. Our second aim was to determine if distal factors (parental acceptance and school environment) act to moderate the relationship between gender diversity and neural correlates of stress (the insula and fusiform gyrus). We hypothesized that these relationships would be weaker with higher parental acceptance and positive perceptions of school environment, and thus be targets for intervention. Finally, we aimed to examine the relationship between gender diversity, parental acceptance, perceptions of school and emotional and behavioral health concerns. We hypothesized that gender diversity would be associated with lower parental acceptance, poorer perceptions of school environment, and elevated mental health problems.

## 2. Materials and methods

### 2.1. Participants

**FIGURE 1:**
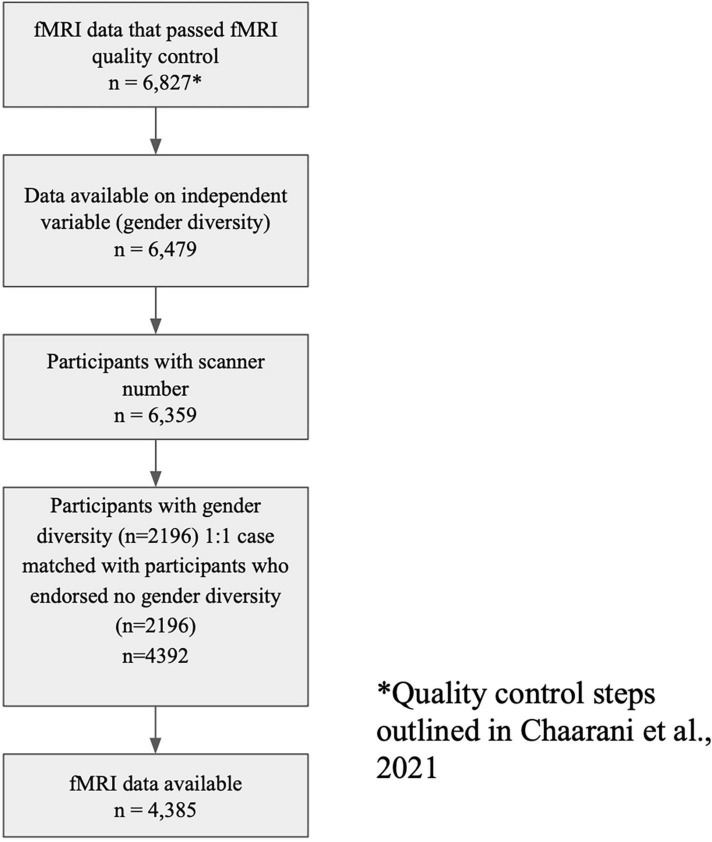
Inclusion criteria and number of participants remaining after each step of exclusion. *Quality control steps outlined Chaarani et al. (2021).

Data from the Adolescent Brain Cognitive Development Study (ABCD)^®^ study, obtained from the National Institute of Mental Health (NIMH) data archive (release 4.0) was used for this project. ABCD is a large, longitudinal study of 11,875 adolescents enrolled at ages 9–10 across the United States. Parent and child participants were primarily recruited through schools, with minimal exclusion criteria (Garavan et al., 2018). All participants provided consent/assent and the University of California San Diego’s Institutional Review Board approved the study protocol. The demographics of the ABCD study participants approximate the demographics of 9–10-year-old youth from the 2015 American Community Survey. ABCD’s inclusion/exclusion flag was utilized to remove subjects who did not pass imaging quality control (Hagler et al., 2019 for more information on ABCD fMRI processing and quality control). To better balance the sample (many participants did not endorse gender diversity), maximize variability associated with gender diversity, and attempt to eliminate confounding that may impact neurobiology, a 1:1 nearest neighbor propensity score without replacement case matching technique was used. This technique used a propensity score estimated utilizing logistic regression of the group (participants that endorsed gender diversity *n* = 2,196 vs. participants that did not endorse any gender diversity; total sample = 4,392) on the covariates which included scanner, age, race, puberty, sex, and highest household education (HHE). See Figure 1 for inclusion criteria and the number of participants remaining after each exclusion.

### 2.2. Behavioral measures

#### 2.2.1. Gender diversity

Gender diversity was measured using four-items (each on a 5-pt scale) that assess felt-gender, contentedness with sex assigned at birth, and gender expression (see Table 2 for gender questions; Potter et al., 2021). A sum score (range 4–20) of the addition of all four items was recoded with higher scores indicating greater gender diversity. Therefore, participants with a score of four endorsed no gender diversity and those who endorsed some amount of gender diversity had scores ranging from 5 to 20. Participants were excluded if they were missing more than two items, for participants missing one to two items, other items were averaged and then used as the value for the missing item(s). Given that many children at this developmental age do not have a clearly defined gender identity or expression, a summary score was used (as opposed to examining the dimensions separately) in order to maximize the variability of the data. Additionally, the items that assess contentedness, gender expression and felt-gender are highly correlated with each other.

**TABLE 2:** Multi-dimensional assessment of gender.

How much do you feel like a < boy/girl >?
How much do you feel like a < girl/boy >?
How much have you had the wish to be a < girl/boy >?
How much have you dressed or acted as a < girl/boy >during play?

#### 2.2.2. Stress-school

The School Environment subscale from the PhenX School Risk and Protective Factors protocol originally derived from the Communities That Care Youth Survey (Arthur et al., 2007) examines youth’s perceptions of their school climate and school engagement. Statements are endorsed on a scale from 1 (*definitely not true)* to 4 (*definitely true).* The items “I feel safe at my school,” “I get along with my teachers,” and “The school lets my parents know when I have done something well” were used in the behavioral analysis as indicators for a latent factor with higher scores indicating more positive perceptions of school environment. The school environment subscale was used as a moderator in the moderation analysis.

#### 2.2.3. Stress-family

The Child Report of Behavior Inventory (CRPBI; Schaefer, 1965; Barber, 1997) is a measure of youth’s perceptions of caregiver acceptance. Higher scores indicate greater warmth/acceptance. Participants report on both the parent or caregiver who is participating in the study (most often biological mothers) and a second caregiver (e.g., grandfather, other mother, father). Mean scores for each caregiver were used in the behavioral analysis as indicators of a latent factor with higher scores indicating greater acceptance. For the moderation analysis, mean scores for each caregiver were averaged and used as a moderator.

#### 2.2.4. Mental health problems

##### 2.2.4.1. Child behavior checklist (CBCL)

The Child Behavioral Checklist (CBCL) is an empirically driven, standardized, dimensional parent-report measure that examines emotional and behavioral problem items (Achenbach and Rescorla, 2001). In the current study, we used raw scores from the broadband Internalizing and Externalizing scales as indicator variables for a latent factor that we labeled mental health problems. Higher scores indicate more problems.

##### 2.2.4.2. Brief problem monitor-youth form (BPM-Y) for ages 11–18

The Brief Problem Monitor-Youth (BPM-Y) is a short-form based on the Youth Self-Report Form, a complement to the CBCL (YSR; Achenbach et al., 2011). The BPM-Y Internalizing and Externalizing raw problem scores were used as indicators for latent mental health problems, with higher scores indicating more problems.

### 2.3. Covariates

Covariates used in this study were used to account for factors associated with gender diversity; neuroimaging signal; and stress. Age in months, pubertal status, and sex assigned at birth have been previously associated with gender diversity in the ABCD study (Potter et al., 2021). Race and highest household education were included to account for effects of systemic racism and discrimination related to income level. Scanner number accounted for differences between scanners across sites. Finally, family ID nested within site was used to account for the sibling relationships in ABCD. All covariates except scanner ID and puberty were reported through the parent-reported demographics survey (Barch et al., 2018). Parent-reported pubertal status was collected with the Pubertal Development Scale (Petersen et al., 1988) which yields five categories (1 = pre-pubertal, 2 = early pubertal, 3 = mid-pubertal, 4 = late-pubertal, and 5 = late-pubertal).

### 2.4. Functional MRI acquisition

The ABCD scanning protocol is harmonized for use across three 3T scanner platforms [Siemens Prisma, General Electric (750) and Phillips] and uses multi-band imaging across 21 sites. The ABCD scan protocol includes collection of structural, diffusion and functional MRI (fMRI; both resting state and task-based fMRI) images. The fMRI acquisitions (2.4 mm isotropic, TR = 800 ms) utilize multiband EPI with slice acceleration factor six. The T1w acquisition (1 mm isotropic) is a 3D T1w inversion prepared RF-spoiled gradient echo scan that uses prospective motion correction (currently only on Siemens and GE scanners). The T2w acquisition (1 mm isotropic) is a 3D T2w variable flip angle fast spin echo scan. The T2w also uses prospective motion correction (but only on Siemens and GE scanners). For further details on the ABCD imaging protocol see Casey et al. (2018) and Hagler et al. (2019).

### 2.5. EN-Back task

The EN-Back task is a working memory task with a block design consisting of two working memory conditions (0-back and 2-back); and two stimuli conditions (emotion faces and places). The task is administered in two runs each containing eight blocks of trials and four 15-s periods containing a fixation cross. There are 160 trials in total with 96 unique stimuli of four types (happy faces, fearful faces, neutral faces, and places). Participants completed the EN-Back in a high spatial and temporal resolution simultaneous multi-slice/multiband echo-planar imaging (EPI) scanner with fast integrated distortion correction. Further information on fMRI quality control and processing pipelines have been previously described by Casey et al. (2018) and Hagler et al. (2019). EN-Back data used in these analyses included individual subject level GLM beta coefficients and s.e.m. (calculated from the ratio of the beta and *t*-statistic) calculated for each voxel and vertex. The faces vs. places contrast (collapsed across working memory conditions) from the baseline neuroimaging session (when youth were ages 9–10) were analyzed. The faces vs. places contrast is activation when viewing emotional faces (happy faces, fearful faces, and neutral faces) minus activation when viewing places stimuli.

### 2.6. Analytic strategy of aim 1—Characterize the association between patterns of brain activation during emotional face stimuli and gender diversity

The first aim of this study was to examine the relationship between gender diversity (collected at the year 1 time point, Mage = 10.93) and patterns of activation (collected at ages 9–10 at the baseline timepoint, Mage = 9.93) while participants were viewing emotion faces (minus activation when viewing places) to investigate if gender diverse youth have patterns of activation similar to other populations that experience stress. Freesurfer’s Permutation Analysis of Linear Models’ (PALM; Winkler et al., 2014). General Linear Model (GLM) was used to generate a cortical and subcortical map that regressed gender diversity (sum score) on the faces minus places contrast (collapsed across working memory load). Permutation testing was utilized to improve reproducibility and robustness of findings. To better balance the sample (many participants did not endorse any gender diversity), maximize variability associated with gender diversity, and attempt to eliminate confounding that may impact neurobiology, we used a 1:1 nearest neighbor propensity score without replacement case matching technique using the MatchIt package (Ho et al., 2011). This technique used a propensity score estimated utilizing logistic regression of the group (participants that endorsed gender diversity *n* = 2,196 vs. participants that did not endorse any gender diversity; total sample = 4,392) on the covariates which included scanner, age, race, puberty, sex, and highest household education (HHE). Covariates used in the fMRI model were from the baseline timepoint. Case matching yielded good balance as evidenced by all the standardized mean differences for the covariates being below 1. Seven participants in the sample were missing data on highest household education (0.1% of the sample prior to case-matching) and 149 (2.3%) were missing parent reported pubertal status. Prior to case matching, missing values for these participants were median imputed based on the participant’s sex and site. Seven more participants were excluded due to not having available fMRI data (final sample = 4,385). Because pubertal status and sex assigned at birth are inextricably linked to the independent variable (gender diversity), and the dependent variable (fMRI activation) these variables were case-matched, but not included as covariates. Although case matching yielded good balance, the standardized mean difference for race and household education were greater than the standardized mean difference of puberty and sex assigned at birth. Further, race and household education do not have a biological basis and are ways in which to categorize how individuals are differentially impacted by social institutions and systems that enact harm due to racism and classism. Thus, race and household education were included as covariates in the model (Buchanan et al., 2021). Scanner number was included as a dummy coded covariate to account for the effects of different scanners on the neuroimaging results. To account for statistical dependency of family structure of participants in the study, PALM’s exchangeability blocks were used, which allows for modeling of the dependence of siblings in the requested 1,000 permutations (Winkler et al., 2015). Gender diversity and all the covariates were mean centered, consistent with PALM program requirements and previous methods (Chaarani et al., 2021). FDR corrected *p*-value maps thresholded at <0.05 were used to determine statistically significant areas of activation.

### 2.7. Aim 2—Examine if stress factors, parental acceptance and school environment, moderate the relationship between gender diversity and heightened activation in the insula and fusiform regions

**FIGURE 2:**
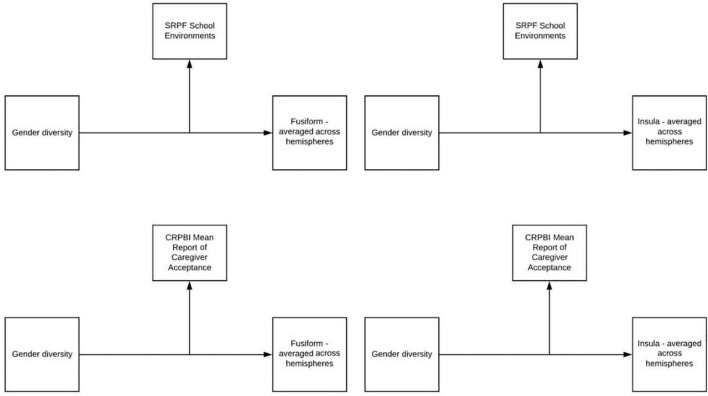
Moderation models.

Four regions of interest were extracted from the task activation map: the fusiform (left and right hemisphere) and the insula (left and right hemisphere) and the ROIs were averaged across hemispheres. Next, four moderation models were run. For the first analysis, youth’s report of their parent’s acceptance and their caregiver’s acceptance were averaged. The average score was used as a moderator to determine if overall parental acceptance moderated the relationship between gender diversity and insula activation. This analysis was repeated but with fusiform activation (averaged across hemispheres) as the dependent variable. In addition to examining the moderating role of parental acceptance, we also investigated if perceptions of school environment moderated the relationship between gender diversity and insula activation. The analysis was then repeated but with the fusiform ROI as the dependent variable. See proposed models in Figure 2.

### 2.8. Strategy of aim 3—Examine the relationship between parental acceptance, perceptions of school environment, gender diversity and mental health symptoms

**FIGURE 3:**
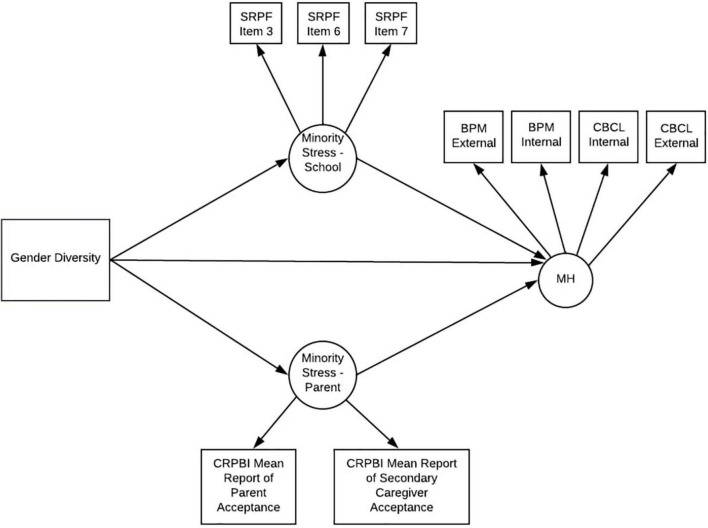
Proposed behavioral model. SRPF, school risk and protective factors; Item 3 = “I get along with my teachers”; Item 6 = “I feel safe at my school”; Item 7 = “The school lets my parents know when I’ve done something well”; BPM, brief problem monitor (ASEBA brief problem monitor—youth form for ages 11–18); CBCL, child behavior checklist [child behavior checklist for ages 6–18 (CBCL/6–18)]; CRPBI, children’s report of parent behavior inventory.

We examined the relationship between gender diversity and mental health problems using a structural equation modeling framework (see Table 3 for proposed stress and mental health indicators and Figure 3 for proposed SEM model). All variables used in the behavioral model were collected at the year 1 time point (Mage = 10.93). Participants used in the fMRI analyses were used in the behavioral analyses. Data were used from the year 1 timepoint when children were ages 10–11 (the participants’ second in-person visit; *n* = 4,385). All behavioral analyses were conducted in R version 4.0.0 (RStudio Team, 2020) using the lavaan package (Rosseel, 2012). Prior to analyzing the path models, measurement models were conducted to determine if better model fit would be achieved through modeling stress as one or two factors (parent-related and school-related stress). To scale the latent factors, the first indicator of each latent factor was fixed to one. To account for the statistical dependency of family, cluster robust standard errors were utilized. To account for missing data and positive skew of some of the indicator variables, Maximum Likelihood Robust estimation was used which allowed for the entire sample of 4,385 to be analyzed. For the SEM model, covariates (sex assigned at birth, race, age in months, and highest household education) were included as exogenous predictors with one-headed paths to each variable in the model.

**TABLE 3:** Latent construct and indicators.

Latent construct	Items/Scale	Measure
School-related stress	Item 3 “I get along with my teachers”	School risk and protective factors Arthur et al. (2007)
	Item 6 “I feel safe at my school”	
	Item 7 “The school lets my parents know when i have done something well”	
Parent-related stress	Mean report of parent by youth	Child report of parent behavior inventory (CRPBI; Schaefer, 1965; Barber, 1997)
	Mean report of secondary caregiver by youth	
Mental health	Parent-reported total externalizing problems	Child behavior checklist total problems Achenbach and Rescorla (2001)
	Parent-reported total internalizing problems	
	Youth-reported total externalizing problems	Brief problem monitor (BPM) total problems Achenbach et al. (2011)
	Youth-reported total internalizing problems	

## 3. Results

### 3.1. Participants

See Table 4 for demographics of participants.

**TABLE 4:** Demographics of participants used in analyses.

Baseline visit demographics			Demographics second in-Person visit (year 1)		
Variable	Number	Percentage	Variable	Number	Percentage
Sex assigned at birth			N/A sex and race remain constant		
Female	2,896	66			
Male	1,489	34			
Race/ethnicity					
White	2,588	59			
Black	470	10.7			
Latino/latine/latinX	818	18.7			
Asian	96	2.2			
Other	413	9.4			
**Highest household education**			**Highest household education**		
<High school diploma	143	3.3	<High school diploma	147	3.4
High school diploma/GED	282	6.4	High school diploma/GED	266	6.1
Some college	976	22.3	Some college	1,002	22.9
Bachelors degree	1,206	27.5	Bachelors degree	1,194	27.2
Post graduate degree	1,757	40.1	Post graduate degree	1,767	40.3
Not answered/Declined to answer/Do not know	21	0.4	Not answered/Declined to answer/Do not know	9	0.2
**Combined income**			**Combined income**		
<50 K	965	22	<50 K	902	20.6
50–100 K	1,176	26.8	50–100 K	1,127	25.7
>100 K	1,949	44.4	>100 K	2,090	47.7
Not answered/Declined to answer/Do not know	295	6.7	Not answered/Declined to answer/Do not know	266	6.1

### 3.2. Results—Aim 1

**FIGURE 4:**
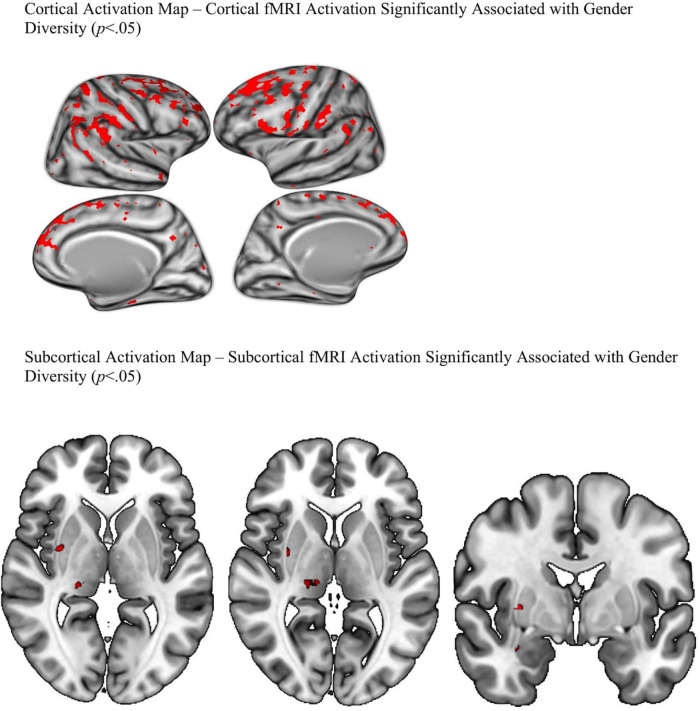
FDR corrected *P*-value maps.

Cortical and subcortical *p*-value maps were generated to model activation patterns of recoded gender diversity on emotional faces (activation when viewing faces—neutral, happy, and fearful, minus activation when viewing places, collapsed across working memory load) and are displayed in Figure 4. On the cortical map, gender diversity was associated with wide-spread greater bilateral activation in task-related areas. Specifically, the fusiform, orbitofrontal (lateral and medial) areas, rostral middle frontal region, occipital and parietal regions. Consistent with our hypothesis, the cortical map showed greater activation of bilateral insula, superior temporal gyrus, and parahippocampal gyrus with more gender diversity when viewing emotional faces compared to places (*p* < 0.05, FDR-corrected). The subcortical map suggested gender diversity associated with right amygdala, as predicted by the stress literature, as well as activation in the right putamen. No differential activation was detected in the dorsal anterior cingulate cortex in either map.

### 3.3. Results—Aim 2

Results from the four moderation models were not significant. Perceptions of the school environment did not moderate the relationship between gender diversity and heightened insula or fusiform activation. Similarly, parental acceptance did not significantly moderate these relationships.

### 3.4. Results—Aim 3

See Table 5 for descriptive statistics and bivariate correlations of the indicators used in aim 2.

**TABLE 5:** Bivariate correlations and descriptive statistics for aim 2.

Indicator variables	Mean (SD)	1	2	3	4	5	6	7	8	9	10	11	12	13	14	15	16	17	18
1. SRPF item 3	3.67 (0.55)	1																	
2. SRPF item 6	3.71 (0.56)	0.34**	1																
3. SRPF item 7	3.20 (0.84)	0.24**	0.26**	1															
4. Gender diversity	18.8 (1.94)	-0.10**	-0.13**	-0.06**	1														
5. Highest household Ed.	3.96 (1.08)	0.06**	0.09**	-0.02*	-0.05**	1													
6. Sex assigned at birth	0.66 (0.47)	0.12**	0.06**	0.07**	0.10**	-0.03	1												
7. Black	0.11 (0.31)	-0.10**	-0.08**	-0.01	0.06*	-0.23**	0.07**	1											
8. Latine/LatinX/Latino	0.19 (0.39)	-0.01	-0.04*	0.01	0.01	-0.31**	0	-0.15**	1										
9. Asian	0.02 (0.14)	0	0.01	0	-0.03	0.09**	0.01	-0.05**	-0.07**	1									
10. Other Race	0.09 (0.29)	-0.03*	-0.04*	-0.01	0.02*	0.02**	0.01	-0.10**	-0.15**	-0.05*	1								
11. Puberty score	2.27 (1)	-0.01	-0.04*	-0.01	0.10**	-0.14**	0.56**	0.19**	0.08**	-0.02	0.01	1							
12. Age in months	131.2 (7.74)	-0.08**	-0.06**	-0.04*	-0.03*	0.01	0.02	0	-0.02*	-0.01	0.02	0.29**	1						
13. BPM-youth externalizing	1.89 (1.92)	-0.30**	-0.21**	-0.19**	0.23**	-0.10**	-0.11**	0.06**	0.02	-0.03*	0.03*	0.01	0.05*	1					
14. BPM-youth internalizing	1.75 (2.08)	-0.18**	-0.28**	-0.17**	0.24**	-0.08**	-0.03*	0.02	0.05*	-0.02*	0.03*	0.06**	0.04*	0.43**	1				
15. CBCL externalizing	3.62 (5.03)	-0.16**	-0.11**	-0.06**	0.06**	-0.09**	-0.11**	0.04*	0.01	-0.06**	0.01	0	0	0.30**	0.14**	1			
16. CBCL internalizing	5.03 (5.40)	-0.10**	-0.10**	-0.04**	0.08**	-0.03	0.01	-0.04**	0.01	-0.05*	0.04*	0.06**	0.02	0.15**	0.27**	0.56**	1		
17. Caregiver acceptance	2.77 (0.31)	0.21**	0.18**	0.22**	-0.13**	0.05*	0.07**	-0.01	-0.04	-0.01	-0.04*	-0.01	0.03*	-0.23**	-0.20**	-0.09**	-0.08**	1	
18. Parental acceptance	2.81 (0.28)	0.26**	0.25**	0.23**	-0.12**	0.09**	0.05**	-0.07**	-0.04*	-0.01	-0.04*	-0.02	0.04	-0.31**	-0.26**	-0.15**	-0.07**	0.39**	1

*p < 0.05, **p < 0.001.

#### 3.4.1. Confirmatory factor analyses

We first examined the fit of a model with one latent stress factor. The overall goodness of fit statistics (as recommended by Hu and Bentler, 1999) for the one factor model indicated poor model fit, χ2 (26) = 1,678.69, *p* < 0.001, RMSEA = 0.13 (90% CI = 0.12, 0.14), SRMR = 0.07, TLI = 0.61, CFI = 0.72. Examination of the factor loadings revealed that parent-reported Internalizing and Externalizing Problems from the Child Behavior Checklist loaded poorly onto the mental health latent factor compared to youth Externalizing and Internalizing Problems (*R* = 0.49; *R* = 0.44, respectively). Dropping the parent-reported indicators from the one factor model improved model fit, χ^2^ (13) = 265.55, *p* < 0.001, RMSEA = 0.07 (90% CI = 0.06, 0.08), SRMR = 0.03, TLI = 0.93, CFI = 0.89.

Next, the fit of a two-factor model (labeled parent- and school-related stress) was analyzed using confirmatory factor analysis. Goodness of fit indices indicated that the two-factor solution provided good fit to the data, χ^2^ (11) = 122.43, *p* < 0.001, RMSEA = 0.05 (90% CI = 0.04, 0.06), SRMR = 0.02, TLI = 0.95, CFI = 0.97. Thus, the two-factor solution was used for the structural equation model.

#### 3.4.2. Structural equation model

**FIGURE 5:**
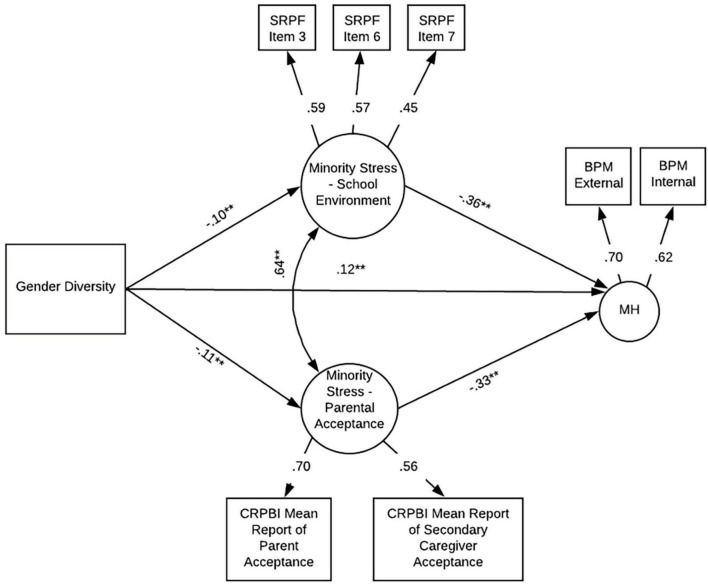
Final model. SRPF, school risk and protective factors; Item 3 = “I get along with my teachers”; Item 6 = “I feel safe at my school”; Item 7 = “The school lets my parents know when I’ve done something well”; BPM, brief problem monitor (ASEBA brief problem monitor—youth form for ages 11–18); CRPBI, children’s report of parent behavior inventory; MH, mental health. **p < 0.001.

Figure 5 shows the model predicting the factor labeled mental health. Fit indices suggest that the model was a good fit to the data χ^2^ (47) = 223.77, *p* < 0.001, RMSEA = 0.03 (90% CI = 0.03, 0.04), SRMR = 0.02, TLI = 0.93, CFI = 0.96. All factor loadings were statistically significant (*p* < 0.001) and ranged from 0.44 to 0.59 for school-stress, were 0.70 and 0.56 for primary and secondary caregiver parental acceptance (respectively), and 0.62 to 0.70 for mental health problems. Examination of the structural model revealed that gender diversity was associated with poorer perceptions of school environment, lower reports of parental acceptance and increased mental health problems. Positive perceptions of school environment and higher parental warmth were associated with reduced mental health problems. To determine if school environment and parental acceptance exert an equal effect on mental health problems, we constrained the paths from school environment to mental health and parental acceptance to mental health. A chi square difference test was significant, χ2 (48) = 187.98, *p* < 0.001, suggesting that the model fit benefits from having separate paths from school environment to mental health problems and parental acceptance to mental health problems.

## 4. Discussion

### 4.1. Conclusion and implications

The current study found that greater gender diversity in a community sample of youth is associated with increased BOLD signal in task-related brain regions. Specifically, the fusiform, orbitofrontal (lateral and medial) areas, rostral middle frontal region, occipital and parietal regions known to be active during recognition, visual/sensory perception, emotion, and memory. Consistent with our hypothesis, gender diversity was also associated with areas commonly activated in populations that experience stress such as the superior temporal gyrus (e.g., De Bellis et al., 2002; Hein and Monk, 2017), parahippocampal gyrus, amygdala and insula (e.g., Hein and Monk, 2017). Inconsistent with our hypothesis, gender diversity was not associated with activation in the dorsal anterior cingulate cortex. This may suggest that the dorsal anterior cingulate cortex is not associated with stress related to gender diversity. An additional reason we may not have found significant findings in the dorsal anterior cingulate is because gender diversity and fMRI imaging data were collected at different time points. The effect may have been stronger had gender diversity and brain imaging been collected at the same time. Our results support our hypothesis that gender diversity is associated with brain activation patterns that are consistent with other types of childhood stress (e.g., Etkin and Wager, 2007; Hein and Monk, 2017; Miller et al., 2020).

Parental acceptance nor youth perceptions of their school environments moderated the relationship between gender diversity and elevated activation in the insula or fusiform. This may be due to several factors. The first is that the school environment and parental acceptance measures ask youth about parental acceptance and their experience at school generally, and not in relation to their gender diversity. It will be important for future studies to replicate this study using measures that specifically assess parental acceptance and school environment as it relates to youth’s gender diversity. Another potential reason that school environment and parental acceptance did not moderate the relationship between gender diversity and elevated activation is that fMRI activation while viewing emotional faces may have been more related to expectation or fear of rejection (a proximal minority stressor) as opposed to more distal stressors like parental acceptance or school environment. Finally, it is possible that the stress factors did not moderate the relationship between gender diversity and task activation due to the developmental age of the participants. Further cumulative experiences of stressful home and school environments may strengthen their modifying effects.

Finally, we found that in a sample with mostly minor levels of gender diversity, this variability is associated with decreased parental acceptance, poorer perceptions of school environment and elevated mental health problems such that the more gender diverse a child is the less parental acceptance they experience, the poorer their perception of school and the more elevated their behavioral and emotional symptoms. Positive perceptions of school environment and higher parental acceptance were associated with fewer behavioral and emotional health problems; and school environment and parental acceptance were found to be separate contributors to behavioral and emotional health problems. This suggests that the more supportive and positive a child’s school environment, the more protective these factors are against the emergence of mental health problems. It is important to note that gender diversity in this study was measured dimensionally and that most participants in the sample do not have a gender minority identity.

The findings of our study are important as they suggest that even minor levels of gender diversity in community youth are associated with markers of stress in the brain. Our behavioral findings provide further support for stress experienced by gender diverse youth by demonstrating the relationship between gender diversity and lower parental acceptance as well as poorer perceptions of the school environment. Our study underscores the importance of policies and legislation to ensure that school environments protect and celebrate gender diverse youth and highlights the importance of funding to provide support to families of gender diverse youth. It is also significant that school and family environments contribute uniquely to outcomes, suggesting multiple gateways for improving outcomes for youth. Promoting acceptance and protection of gender diversity within school and family environments may have important preventative effects. Extant literature suggests a relationship between minority stressors such as gender-based victimization, bullying, lack of parental or familial support, and suicidal behaviors (e.g., Bochicchio et al., 2021). Further, minority stress is associated with behavioral dysregulation, an important mediator of the relationship between minority stress and elevated suicidal ideation (e.g., Drescher et al., 2023). Although these studies were conducted with youth and adults with a gender minority status, our findings suggest a broader, generalizable relationship that extends to youth who endorse some level of gender diversity.

### 4.2. Limitations and future directions

#### 4.2.1. Measurement considerations

It is important to consider the limitations of this study when interpreting the results. A notable limitation of the current study is that the brain analyses and gender diversity data were collected at different time points. This is due to the ABCD study design as gender diversity was not measured at the study baseline visit (Potter et al., 2021). However, we believe, if anything, that this underestimates the relationships we report. Stress related to gender diversity-based bullying or victimization is likely not as prevalent at ages 9 and 10. Existing developmental literature suggests that teasing and victimization related to gender diversity emerges in early adolescence. Early adolescence is a time of “gender intensification” where peers exert pressure to conform to gender stereotypes (Hill and Lynch, 1983) and children who do not follow gender norms are more likely to be rejected or teased (e.g., Thorne, 1993; Martin and Ruble, 2010). Nevertheless, due to the reverse temporality of gender diversity, results of the study should be interpreted cautiously. Despite the limitation of the temporality of the measures, gender diversity within the sample appears to be stable across time. For instance, 77.8% of youth with data at year 1 (when gender diversity data was first collected) and year 2 either became more gender diverse or maintained the same level of gender diversity. While this was a cross-sectional study that used data from two time points, it is noteworthy that the data used is part of an ongoing study that will allow for future longitudinal assessment. It will be important for future studies to examine longitudinal associations between gender diversity, stress and fMRI activation during an emotion faces task.

Another important limitation is that the stressors in our behavioral model were not specifically assessing parental acceptance and perceptions of school environment as they relate to gender diversity. Although our findings suggest that there is a relationship between gender diversity, mental health, school perceptions, and parental acceptance in the behavioral model, our findings do not indicate that these outcomes are caused by youth gender diversity. Further studies should examine the relationship between school perceptions and parental acceptance related to gender diversity and mental health outcomes.

#### 4.2.2. Intersectionality

Our study did not examine intersecting identities such as race, income, or ability. Intersecting bias and prejudice toward gender diverse youth may confer additional or unique risk and protective factors. Future research should examine how gender diversity-related stress uniquely impacts the neurobiology and mental health of youth with intersecting identities. Another notable limitation is that we did not examine sex differences in this study. There is some research that suggests males assigned at birth are more heavily penalized for gender diversity, specifically gender non-conformity, compared to females (e.g., van Beusekom et al., 2020). Future research should examine sex differences in gender diversity-related stress using both behavioral and fMRI methods.

## 5. Conclusion

Despite the above limitations, our study has important implications for our understanding of the relationship between parental acceptance, perceptions of school environment, brain function, and mental health among youth who endorse some level of gender diversity. This is the first study to examine the neural underpinnings of gender diversity and stress in a community sample of children. Our results suggest that gender diversity is associated with patterns of brain activity that are consistent with other populations who experience stress—a finding that may help to understand the consequences of expecting discrimination and/or peer victimization related to gender. While parental and school stressors did not moderate heightened fMRI activation, the current study contextualized the heightened fMRI activation by demonstrating a relationship between gender diversity, peer and family related stress, and elevated behavioral and emotional problems. Further, our findings suggest a relationship between gender diversity, family and school stressors, neurobiology and mental health even among youth who are not non-binary or transgender. Our fMRI and behavioral findings suggest that there are mental health consequences and neural underpinnings of external stress factors experienced by gender diverse youth. Our findings underscore the importance of creating school and family environments that serve as a buffer against gender diversity-related stress.

## Data availability statement

Publicly available datasets were analyzed in this study. This data can be found here: doi: 10.15154/1523041..

## Ethics statement

This study involves human participants and was reviewed and approved by the Appropriate Institutional Review Boards. Written informed consent to participate in this study was provided by the participants’ legal guardian and youth provided written assent.

## Author contributions

HL performed the statistical analysis and wrote the first draft of the manuscript. MA provided mentorship and technical support in the creation of the figures. BC provided technical support and mentorship of the fMRI statistical analyses. AP and SD contributed to the conception and design of the study and wrote sections of the manuscript. AP was the senior mentor on the project. All authors contributed to manuscript revision, read, and approved the submitted version.
